# The diagnostic value and efficacy evaluation of lung ultrasound score in neonatal respiratory distress syndrome: a prospective observational study

**DOI:** 10.3389/fped.2025.1500500

**Published:** 2025-01-30

**Authors:** Jian Dong, Yuhong Deng, Jin Tong, Tingting Du, Wenguang Liu, Yan Guo

**Affiliations:** ^1^ Department of Ultrasound Medicine, The First Affiliated Hospital of Shihezi University, Shihezi, Xinjiang, China; ^2^ Department of Research, The First Affiliated Hospital of Shihezi University, Shihezi, Xinjiang, China; ^3^ Department of Pediatrics, The First Affiliated Hospital of Shihezi University, Shihezi, Xinjiang, China

**Keywords:** neonatal respiratory distress syndrome, pulmonary surfactant, neonatal lung ultrasonography score, chest x-ray, grade diagnoses

## Abstract

**Objective:**

To evaluate the diagnostic efficacy and determine the optimal cut-off values of lung ultrasound score for diagnosing neonatal respiratory distress syndrome and its accuracy in assessing the efficacy of neonatal respiratory distress syndrome.

**Method:**

This prospective study included 100 neonates with suspected neonatal respiratory distress syndrome. Each patient underwent both the 14-zone and 12-zone lung ultrasound methods, as well as a chest x-ray, performed after birth and before initiating drug treatment. Surfactant replacement therapy was administered to patients who were diagnosed with neonatal respiratory distress syndrome and met the criteria for medication. Lung ultrasound was conducted and recorded at the 24th hour, the 48th hour, the 72nd hour, and the 7th day after drug administration. ROC curve analysis, Kappa statistics, and ANOVA were utilized to identify the optimal cut-off values for the lung ultrasound scores in diagnosing neonatal respiratory distress syndrome.

**Results:**

89 neonates were diagnosed with respiratory distress syndrome, of whom 64 received surfactant replacement therapy. The mean scores of 12-zone lung ultrasound score, 14-zone lung ultrasound score, and chest x-ray score are 18.22 ± 7.15, 38.92 ± 9.69, and 2.15 ± 0.97, respectively. The diagnostic AUC for the 12-zone lung ultrasound score is 0.84 (95% CI: 0.73–0.95), with an optimal cut-off value of 13.5 for diseased vs. not diseased, while the AUC for the 14-zone lung ultrasound score is 0.88 (95% CI: 0.76–0.99), with an optimal cut-off value of 34 for diseased vs. not diseased. There is significant concordance between the neonatal lung ultrasonography scores and the chest x-ray score for diagnosis respiratory distress syndrome (*P* < 0.01). The optimal cut-off values for the grading diagnosis of neonatal respiratory distress syndrome using the 14-zone lung ultrasound score are identified as 36.5, 40.5, and 44.5. The 12-zone lung ultrasound score does not have a significant difference between the 12th hour after receiving surfactant replacement therapy and the 48th hour after treatment (*P* = 0.08). All other comparisons demonstrated significant differences.

**Conclusion:**

The 14-zone lung ultrasound score demonstrates higher diagnostic efficacy in diagnosing neonatal respiratory distress syndrome and can accurately evaluate the early efficacy of surfactant replacement therapy in neonates.

## Introduction

Neonatal respiratory distress syndrome (NRDS) is a group of disorders caused by immature lung development or a lack of pulmonary surfactant (PS), resulting in the formation of eosinophilic transparent membranes and diffuse lung damage in the respiratory bronchioles and below (1–3). Neonates often present with cyanosis, progressive dyspnoea, and even respiratory failure, accompanied by intercostal and subcostal retractions and expiratory moaning (4). The incidence of NRDS ranges from 1.72% to 8.2%, and it is one of the leading causes of early mortality among neonatal respiratory diseases worldwide (5, 6). Early diagnosis and timely treatment of NRDS play a key role in reducing complications and improving prognosis.

NRDS is primarily diagnosed through a combination of clinical manifestations, radiologic findings, and arterial blood gas analysis. Chest x-ray (CXR) remains the most commonly used imaging tool to diagnose NRDS. However, CXR imaging of NRDS lacks specificity, and various lung diseases may present similar reticular shadows or “white lung” phenomena, which may interfere with the diagnosis of NRDS (7). Neonates are at the peak of growth and development, which makes them more sensitive to ionizing radiation and radiation damage, limiting the use of CXR for repeated examinations and dynamic monitoring of the course of RDS in neonates (8, 9). Additionally, cumulative radiation exposure may elevate the risk of genetic mutation, gonadal damage, and thyroid cancer in neonates (10, 11). Lung ultrasound (LUS) has been gradually involved in the diagnosing and grading of NRDS due to its advantages of being accurate, rapid, radiation-free, and repeatable recently (12, 13). Relevant guidelines have recognized LUS as an important diagnostic imaging method for neonatal lung disease, and higher sensitivity and specificity have been shown in some studies. Its diagnostic value may surpass that of the traditional CXR examinations (14–16).

Replacement therapy with exogenous PS remains an important treatment for NRDS. Early administration of PS reduces the requirement for mechanical ventilation, pneumothorax occurrence, and mortality in neonates (17). The neonatal lung ultrasonography score (nLUS) is strongly associated with neonatal oxygenation status and can accurately determine whether neonates with NRDS under continuous positive airway pressure ventilation require PS replacement therapy (18, 19). Studies advocate for nLUS to be used for semi-quantitative evaluation of lung ventilation to guide the diagnosis of respiratory diseases. Current research evaluating NRDS primarily utilizes the six-zone, ten-zone, and twelve-zone lung ultrasound methods. Studies related to the 14-zone lung ultrasound score (nLUS_14_) is limited, and there is relatively little comparative research on the diagnosis and grading of NRDS between the 12-zone lung ultrasound score (nLUS_12_) and nLUS_14_.

This study compared the diagnostic accuracy of nLUS_12_, nLUS_14_, and CXR in neonates with suspected NRDS. The function of LUS in diagnosing and grading NRDS was investigated, as well as the value of nLUS_12_ and nLUS_14_ in PS replacement therapy.

## Materials and methods

One hundred neonates with highly suspected NRDS in the NICU of the First Affiliated Hospital of Shihezi University from January 2022 to April 2023 were prospectively and consecutively included in this study. All participants underwent LUS and CXR examinations, and of the diagnosed patients, 64 neonates underwent PS replacement therapy for respiratory distress. Inclusion criteria comprised neonates with a gestational age of 24–36 weeks who were treated with nasal continuous positive airway pressure for respiratory distress. The diagnostic criteria for NRDS used in this study were: (1) clinical manifestation: progressive tachypnea, expiratory grunting, subcostal retractions, and cyanosis; (2) chest x-ray abnormalities: air bronchogram signs, dense B-line, ground-glass opacities, or “white lungs”; (3) arterial blood gas analysis: hypoxia, hypercapnia, and oxygen tension/fraction of inspired oxygen ratio PaO_2_/FiO_2_ ≤ 26.7 kPa (17, 20, 21). Exclusion criteria comprised intubation or receiving PS treatment before imaging, neonatal air leak syndrome, chromosomal abnormalities, sepsis, and congenital lung diseases. The study was approved by the Medical Ethics Committee of the First Affiliated Hospital of Shihezi University (No. KJ2022-317-01), and the guardians of the neonates signed a written informed consent. This study strictly adhered to the principles outlined in the Declaration of Helsinki.

### Ultrasound detection methods and nLUS

This study adhered to published guidelines and relevant normative requirements for simultaneous nLUS_12_ and nLUS_14_ in neonates (22, 23). Bedside LUS was performed using a Samsung HM70 portable color Doppler ultrasound device equipped with a high-frequency (10–15 MHz) transducer. The examinations were conducted by two sonographers with over 10 years of qualification and rich experience in pediatric LUS diagnosis. They independently performed the ultrasound sweeps and image analysis simultaneously while blinded to the clinical data of the neonates. Mean B-lines were calculated if the ultrasound window covered more than one intercostal space. The most severe ultrasound manifestations in a given area were scored. Considering that the US inspection results are closely related to the operator's experience and level. When the difference in scores between two doctors does not exceed 5 points, take the average of the rating results of two sonographers. If the difference in scores between two sonographers is significant or even exceeds 5 points, a senior pediatric ultrasound specialist with more than 15 years of experience performed an additional blinded ultrasound examination to provide the nLUS. This study utilized the intraclass correlation coefficients (ICC) to determine the inter-operator variability of ultrasound diagnostic results between two sonographers.

Neonates were scanned in supine, lateral, and prone positions while at rest. Lungs were divided into six regions: anterior-superior, anterior-inferior, axillary-superior, axillary-inferior, posterior-superior, and posterior-inferior, resulting in a total of twelve regions bilaterally. The LUS_14_ was based on the LUS_12_ by adding bilateral lung base regions to be swept with the bilateral rib arch as the boundary. When measuring each zone in supine or lateral position, starting from the zone centerline, the probe takes a longitudinal section and slides outward to the boundary line, returns to the centerline, slides inward to the boundary line, and then returns to the centerline. For the scanning of the four zones of the chest after the prone position, the main approach is to scan from the scapular line to the posterior axillary line.

nLUS_12_ assigns a maximum score of three points and a minimum score of zero points for each region, with 12 regions and a total score totaling thirty-six points. The specific scoring criteria are as follows: (1) Zero points: the presence of A-lines only; (2) One point: the presence of A-lines in the upper part of the lungs, presence of fused B-lines or at least three B-lines in the lower part of the lungs; (3) Two points: the presence of fused B-lines; and (4) Three points: abnormal pleural lines and presence of solid lung lesions (18, 24).

nLUS_14_ assigns a maximum score of five points and a minimum score of zero points for each region, with 14 regions and a total score totaling seventy points. The specific scoring criteria are as follows: (1) Zero point: predominantly A-lines, with scattered (<3) B-lines; (2) One point: scattered B-lines (≥3), no fused B-lines; (3) Two points: dense B-lines, with partially fused B-lines; (4) Three points: fused B-lines; (5) Four points: abnormal pleural lines, with subpleural lung solid lesions; and (6) Five points: abnormal pleural lines, with extensive lung solid lesions (25).

In this study, CXR was performed and scored after LUS examination and prior to PS replacement therapy. A pediatric radiologist with more than 10 years of experience performed CXR photography by using a transportable x-ray machine, GE TMX+ (General Electric, Boston, MA, USA), and Agfa CR 30-X computed radiography imaging system (Agfa-Gevaert, Mortsel, Belgium). Image quality was improved and CXR scoring was performed by pre-processing work such as noise suppression and contrast enhancement.

The CXR classified each region into four grades: (1) Grade I: both lungs were slightly less inflated, with reduced translucency, and fine granular densification was seen in the lung fields. (2) Grade II: the translucency of both lungs is further reduced, and there is ground-glass-like change, with evenly distributed fine granular densification and bronchial insufflation in the lung fields, while the diaphragmatic surface of the heart margin is still clear. (3) Grade III: the fine granular hyperintense shadows in the lungs are fused and enlarged, with blurred edges, increased density in the lung fields, markedly reduced translucency, blurred cardiac margins, and bronchial congestion signs are more pronounced. (4) Grade IV: The uniformly increased density of both lung fields is “white lung”, and the heart edge of the diaphragm disappears completely (26).

### Pulmonary surfactant replacement therapy and examination

This study performed PS replacement therapy on some neonates diagnosed with NRDS. This study administered poractant-α to neonates with gestational age less than 26 weeks and respiratory oxygen concentration (FiO_2_) greater than 30% or to those with a gestational age greater than 26 weeks and FiO_2_ greater than 40%. Positive end-expiratory pressure (PEEP) greater than or equal to 5.0 cmH_2_O is also one of the criterion for exogenous PS replacement therapy (27). All neonates who require PS replacement therapy received surfactants through the endotracheal intubation technique. The initial dose administered was 200 mg/kg poractant-α. At the 24th hour, the 48th hour, the 72nd hour, and the 7th day after PS administration, nLUS_12_ and nLUS_14_ were performed simultaneously on the patient. To minimize the risk of cancer in neonates and adhere to ethical requirements, this study only conducted one CXR examination after birth and prior to PS replacement therapy. CXR examinations were not performed dynamically after PS treatment.

### Statistical analysis

Statistical analyses were conducted using SPSS version 26.0. Quantitative data that conformed to normal distribution were expressed as mean plus or minus standard deviation $\left(\bar{X}\pm s\right)$, and non-normally distributed data were expressed as median and quartiles. Clinical baseline data between NRDS and non-NRDS groups were analyzed using an unpaired *t*-test. Correlation analyses were conducted using Spearman's test. The accuracy of nLUS and CXR scores for the diagnosis and grading scores of NRDS was compared by plotting the Receiver operating characteristic (ROC) curve and calculating the corresponding areas under the ROC curve (AUC). The optimal cut-off point was searched to determine the best threshold for NRDS diagnosis and PS administration or not. The diagnostic consistency of nLUS and CXR was evaluated by the Kappa test to predict the diagnostic efficacy of nLUS in diagnosing NRDS. Differences in nLUS at different time points after PS treatment were evaluated using analysis of variance (ANOVA). Kappa values > 0.75 were considered indicative of good consistency, and *P* < 0.05 was considered to be statistically significant.

## Results

### Baseline characteristics

One hundred neonates with suspected NRDS were included in this study, of whom 89 were diagnosed with NRDS. The clinical baseline characteristics of the included neonates are presented in Table 1. There are 46 males and 54 females, with a mean gestational age of 30.34 ± 2.89 weeks, a median postnatal age of 4.82 (interquartile range: 4.58–5.07) hours, and a mean birth weight of 1,868.65 ± 317.66 g. There are no significant differences in sex, gestational age, postnatal age, and birth weight between the NRDS and non-NRDS groups (*P* > 0.05).

**Table 1:** Characteristics of the patients.

Characteristics	Neonates included	Neonates with RDS	Neonates without RDS
	*n* = 100	*n* = 89	*n* = 11
Gender (male/female)	46/54	41/48	5/6
Gestational age (week)	30.34 ± 2.87	30.11 ± 2.83	32.18 ± 2.75
Postnatal age (hour)	4.82 (4.58, 5.07)	4.83 (4.56, 5.01)	4.66 ± 0.72
Delivery mode (Eutocia/Cesarean delivery)	42/58	36/53	6/5
Weight (gram)	1,868.65 ± 317.66	1,850.15 ± 321.67	2,018.36 ± 246.43
PaO_2_ (mmHg)	73.36 ± 10.12	72.15 ± 9.89	83.18 ± 6.26
FiO_2_ (%)	42.13 (40.36, 43.90)	43.10 (41.24, 49.96)	34.27 ± 5.22
PaO_2_/FiO_2_	1.86 ± 0.57	1.78 (1.67, 1.89)	2.49 ± 0.46
PEEP (cmH_2_O)	5.05 ± 0.43	5.13 ± 0.47	4.42 ± 0.25
LUS inspection time (hour)	3.47 ± 0.73	3.23 (3.06, 4.11)	5.36 ± 0.62
CXR inspection time (hour)	4.73 ± 0.54	4.57 (4.32, 5.01)	6.12 (5.58, 7.07)

Values were mean ± SD, and median (interquartile range). PaO_2_, partial pressure of carbon dioxide; FiO_2_, inspiratory oxygen concentration; PEEP, positive end-expiratory pressure; LUS, lung ultrasound; CXR, chest x-ray.

### Diagnostic accuracy analysis of nLUS and CXR scores

**Figure 1:**
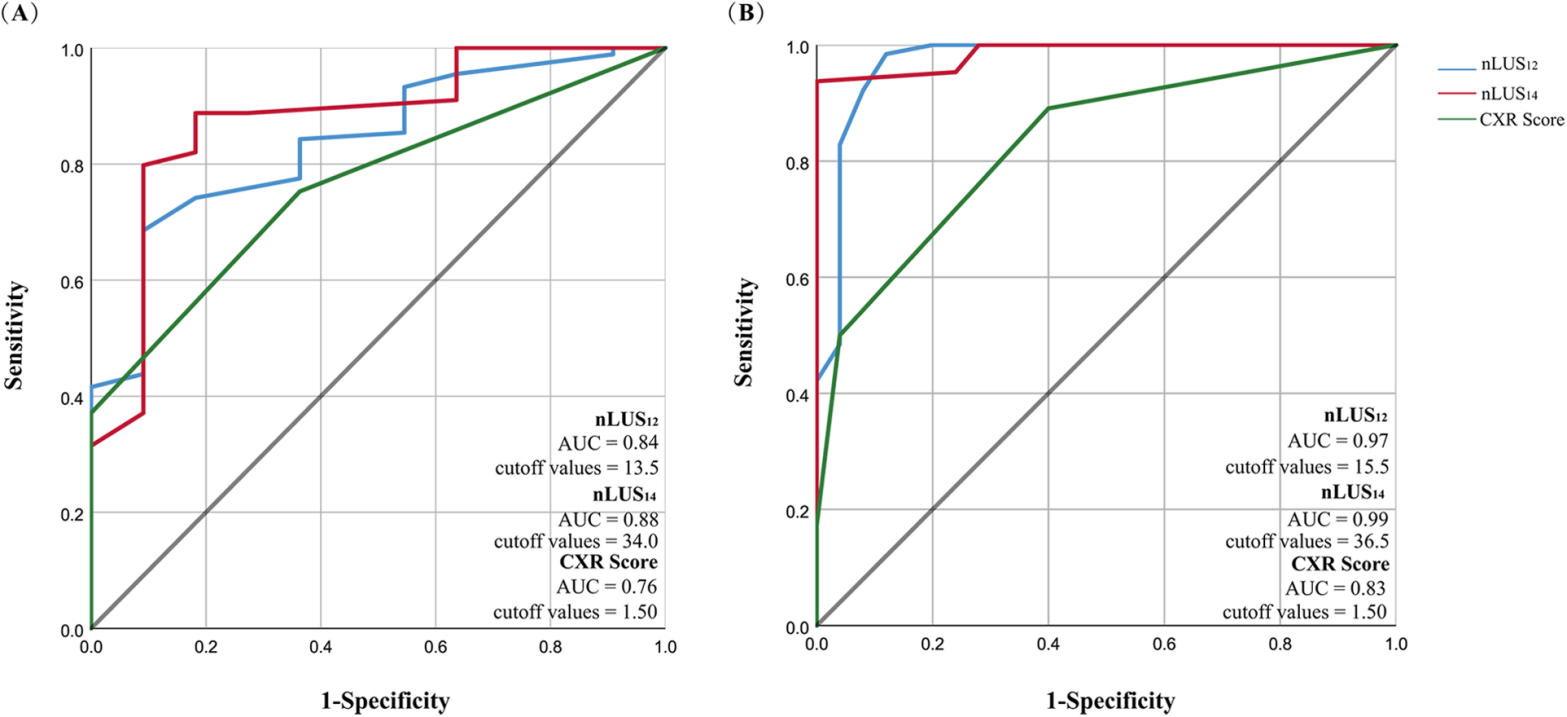
Nlus and CXR score predict the diagnostic value of NRDS and whether PS replacement therapy is needed. **(A)** Diagnostic accuracy analysis of nLUS and CXR score. **(B)** nLUS and CXR scores for assessing the need for PS treatment.

The LUS and CXR scores performed by the subjects within 9 h after birth and prior to PS replacement therapy are shown in Table 2 and Figure 1A. The ICC between the two sonographers was 0.87 (95% CI: 0.84–0.90). The mean scores for nLUS_12_, nLUS_14_, and CXR score are 18.22 ± 7.15, 38.92 ± 9.69, and 2.15 ± 0.97, respectively. No significant differences are observed among the three groups (*P* > 0.05). Spearman's correlation analysis reveals that CXR score is positively correlated with nLUS_12_ and nLUS_14_ (*r* = 0.75, *P* < 0.01. *r* = 0.71, *P* < 0.01). CXR score is significantly negatively correlated with the partial pressure of carbon dioxide (PaO_2_) and PaO_2_/FiO_2_ ratio (*r* = −0.91, *P* < 0.01. *r* = −0.94, *P* < 0.01). CXR score has a significant positive correlation with FiO_2_ (*r* = 0.93, *P* < 0.01).

**Table 2:** Diagnostic accuracy of NRDS for nLUS and CXR score.

	Neonates with RDS	Neonates without RDS	Neonates included						
	*n* = 89	*n* = 11	*n* = 100						
	Score	Score	Score	AUC	*P* value	95% CI	Cutoff values	SEN	SPE
nLUS_12_	19.18 ± 6.70	10.45 ± 6.02	18.22 ± 7.15	0.84	<0.01	0.73–0.95	13.50	0.91	0.64
nLUS_14_	40.54 ± 8.43	25.82 ± 9.51	38.92 ± 9.69	0.88	<0.01	0.76–0.99	34.00	0.94	0.91
CXR Score	2.25 ± 0.97	1.36 ± 0.50	2.15 ± 0.97	0.76	<0.01	0.64–0.88	1.50	0.88	0.64

Values were mean ± SD, and median (interquartile range). nLUS_12_, 12-zone lung ultrasound method; nLUS_14_, 14-zone lung ultrasound method; CXR score, chest x-ray score.

The diagnostic sensitivity (SEN) of nLUS_12_ is 0.91, specificity (SPE) is 0.64, accuracy (ACC) is 0.88, positive predictive value (PPV) is 0.96, and negative predictive value (NPV) is 0.44. The ROC curve is plotted, the AUC of nLUS_12_ is 0.84 (95% CI: 0.73–0.95), and the optimal cut-off value identifies as 13.5. The diagnostic SEN for nLUS_14_ is 0.94, SPE is 0.91, ACC is 0.94, PPV is 0.99, and NPV is 0.67. Plotting the ROC curve, the AUC for nLUS_14_ is 0.88 (95% CI: 0.76–0.99), and the optimal cut-off value identifies as 34. The diagnostic SEN for the CXR score is 0.88, SPE is 0.64, ACC is 0.85, PPV is 0.96, and NPV is 0.37. Plotting the ROC curve, the AUC for the CXR score is 0.76 (95% CI: 0.64–0.88) (see Table 2). There is a significant difference in diagnostic consistency among nLUS_12_, nLUS_14_, and the CXR score. There is good consistency between nLUS_12_ and the CXR score (Kappa = 0.78, *P* < 0.01) There is moderate consistency between nLUS_14_ and the CXR score (Kappa = 0.53, *P* < 0.01).

### nLUS judgment of CXR grading diagnostic score thresholds

**Figure 2:**
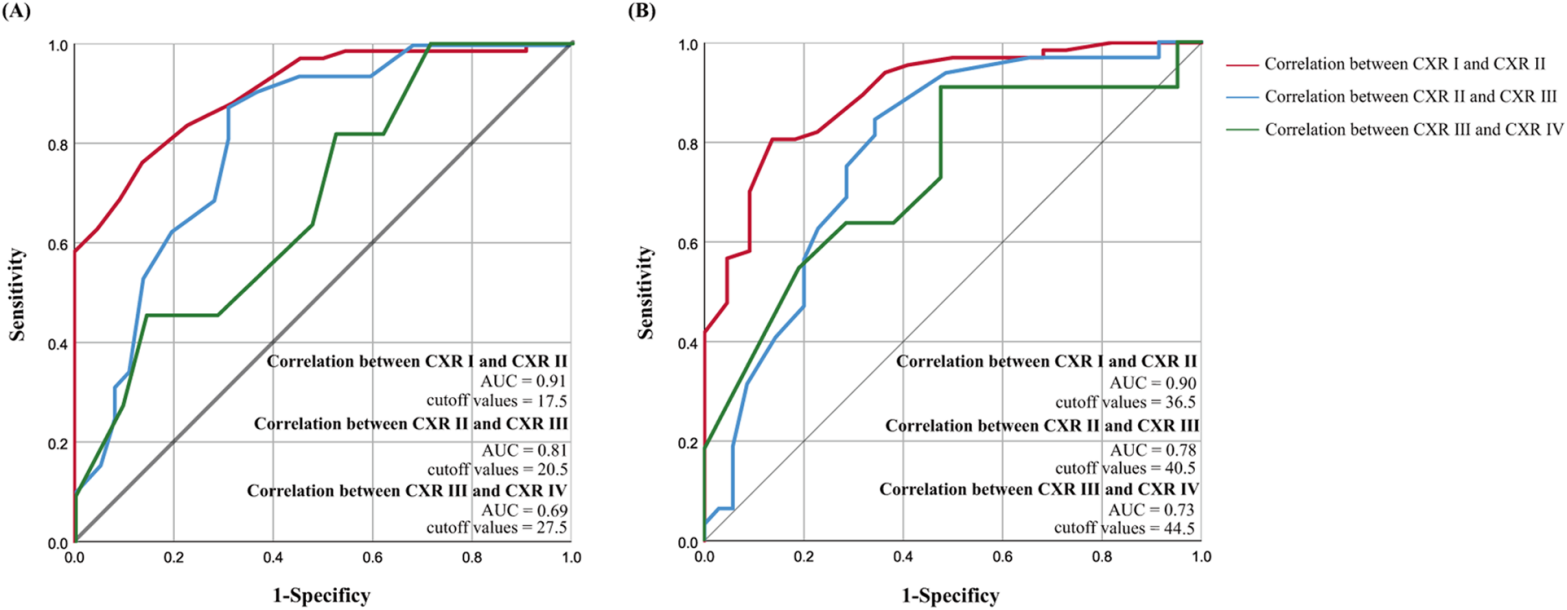
Nlus determines the threshold for CXR grading diagnosis score. **(A)** nLUS_12_ determines the threshold for CXR grading diagnosis score. **(B)** nLUS_14_ determines the threshold for CXR grading diagnosis score.

The relevant data for nLUS_12_, LUS_14_, and the CXR score are presented in Table 3. In nLUS_12_, the optimal cut-off value between CXR grade I and CXR grade II is 17.5, with AUC is 0.91. The optimal cut-off value between CXR grade II and CXR grade III is 20.5, with AUC is 0.81. The optimal cut-off value between CXR grade III and CXR grade IV is 27.5, with AUC is 0.69 (see Table 4, Figure 2A).

**Table 3:** Scoring results of NRDS patients with different CXR grades.

	CXR score				
	Grade I	Grade Ⅱ	Grade Ⅲ	Grade Ⅳ	Overall
	*n* = 22	*n* = 34	*n* = 22	*n* = 11	*n* = 89
PaO_2_ (mmHg)	83.09 ± 4.10	75.09 ± 3.73	65.00 ± 3.59	55.45 ± 5.57	73.36 ± 10.12
FiO_2_ (%)	34.18 ± 1.92	39.24 (38.45, 40.02)	50.36 ± 4.23	58.36 ± 5.26	42.13 (40.36, 43.90)
PaO_2_/FiO_2_	2.44 ± 0.19	1.92 ± 0.17	1.30 ± 0.13	0.96 ± 0.16	1.86 ± 0.57
PEEP (cmH_2_O)	4.72 ± 0.35	5.21 ± 0.64	5.28 ± 0.64	5.39 ± 0.81	5.13 ± 0.47
nLUS_12_	8.86 ± 4.96	15.62 ± 5.01	20.86 ± 3.75	21.00 (16.08, 25.92)	19.18 ± 6.70
nLUS_14_	28.82 ± 5.95	37.32 (34.94, 39.71)	41.86 ± 4.82	46.27 ± 8.05	40.54 ± 8.43

Values were mean ± SD, and median (interquartile range). PaO_2_, partial pressure of carbon dioxide; FiO_2_, inspiratory oxygen concentration; PEEP, positive end-expiratory pressure; nLUS_12_, 12-zone lung ultrasound method; nLUS_14_, 14-zone lung ultrasound method; CXR score, chest x-ray score.

**Table 4:** Nlus judgment of CXR grading diagnostic score thresholds.

nLUS	CXR score	Cutoff values	AUC	95% CI	SEN	SPE	ACC	PPV	NPV
nLUS_12_	CXR grade I—CXR grade II	17.5	0.91	0.84–0.97	1.00	0.81	0.85	0.63	1.00
	CXR grade II—CXR grade III	20.5	0.81	0.71–0.91	0.74	0.91	0.82	0.89	0.77
	CXR grade III—CXR grade Ⅳ	27.5	0.69	0.50–0.88	0.82	0.45	0.70	0.75	0.96
nLUS_14_	CXR grade I—CXR grade II	36.5	0.90	0.83–0.97	0.95	0.85	0.88	0.68	0.98
	CXR grade II—CXR grade III	40.5	0.78	0.67–0.89	0.56	0.88	0.72	0.70	0.71
	CXR grade III—CXR grade Ⅳ	44.5	0.73	0.54–0.93	0.50	0.91	0.64	0.92	0.48

nLUS_12_, 12-zone lung ultrasound method; nLUS_14_, 14-zone lung ultrasound method; CXR score, chest x-ray score.

In nLUS_14_, the optimal cut-off value between CXR grade I and CXR grade II is 36.5, with AUC is 0.90. The optimal cut-off value between CXR grade II and CXR grade III is 40.5, with AUC is 0.78. The optimal cut-off value between CXR grade III and CXR grade IV is 44.5, with AUC is 0.73 (see Table 4, Figure 2B).

### nLUS and CXR score to assess the need for PS treatment

Sixty-four neonates with confirmed NRDS received PS replacement therapy. The mean scores for nLUS_12_, nLUS_14_, and CXR score of all neonates treated with PS are 19.18 ± 6.70, 40.54 ± 8.43, and 2.25 ± 0.97, respectively. The accuracy of nLUS_12_ and nLUS_14_ are significantly higher than that of the CXR score (*P* < 0.01), whereas no significant difference is observed between the nLUS_12_ and nLUS_14_ (*P* = 0.52). Plotting the ROC curve, the AUC of nLUS_12_ is 0.97 (95% CI: 0.93–1.00), and the optimal cut-off value for acceptance or non-acceptance of PS treatment is 15.5. The AUC of nLUS_14_ is 0.99 (95% CI: 0.97–1.00), and the optimal cut-off value for acceptance or non-acceptance of PS treatment is 36.5. The best cut-off value is 36.5. The AUC of the CXR score is 0.83 (95% CI: 0.74–0.92) (see Table 5, Figure 1B).

**Table 5:** Assessment of whether NRDS patients should receive PS replacement therapy in pairs nLUS and CXR score.

	Score	AUC	*P* value	95% CI	Cutoff values
nLUS_12_	19.18 ± 6.70	0.97^a^	<0.01	0.93–1.00	15.50
nLUS_14_	40.54 ± 8.43	0.99^b^	<0.01	0.97–1.00	36.50
CXR score	2.25 ± 0.97	0.83	<0.01	0.75–0.92	1.50

Values were mean ± SD, and median (interquartile range). nLUS_12_, 12-zone lung ultrasound method; nLUS_14_, 14-zone lung ultrasound method; CXR score, chest x-ray score.

^a^ nLUS_12_ vs. CXR score, *P* < 0.01.

^b^ nLUS_14_ vs. CXR score, *P* < 0.01.

### nLUS evaluation of PS treatment efficacy

**Figure 3:**
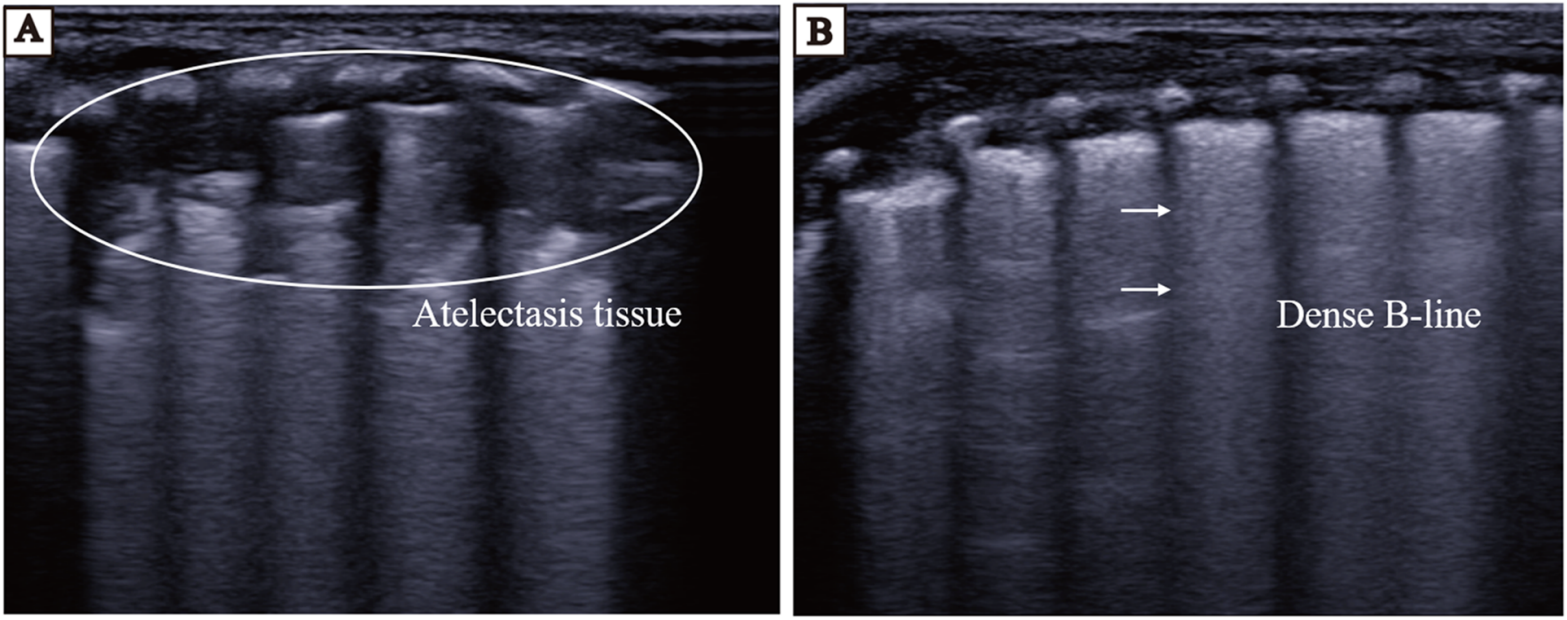
LUS of neonates before and 24 h after PS replacement therapy. **(A)** LUS before the neonates receives PS treatment. The continuity of the pleural line is interrupted, and large areas of atelectasis echo can be seen below the pleural line, with bronchial inflation sign visible inside. **(B)** LUS of the neonates 24 h after receiving PS treatment. Loss of atelectasis and appearance of dense B-line in lung tissue.

**Figure 4:**
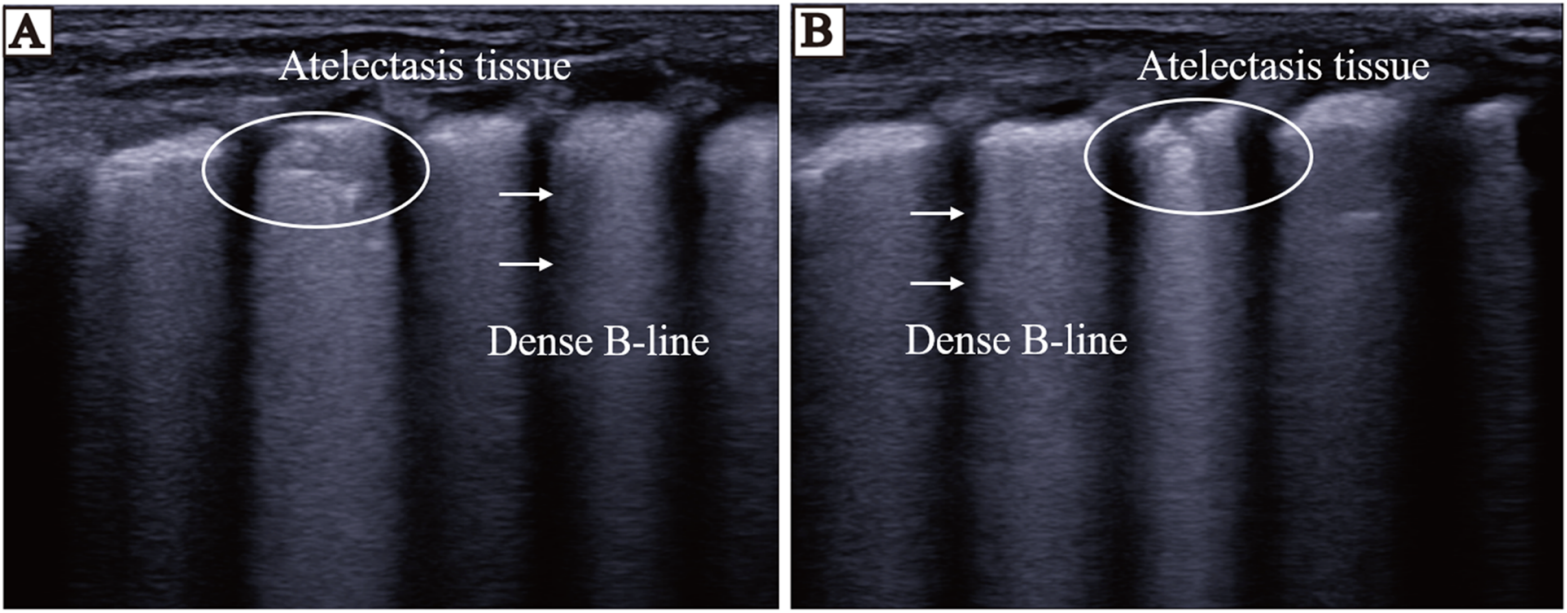
LUS of neonates before and 72 h after PS replacement therapy. **(A)** LUS before the neonates receives PS treatment. Thickening of pleural line, disappearance of A-line, visible dense B-line, echoes of atelectasis in some areas below pleural line, and bronchial inflation sign visible inside. **(B)** LUS of the neonates 72 h after receiving PS treatment. The area below the pleural line shows atelectasis, and the echogenicity of lung tissue is significantly reduced after treatment.

The nLUS results for NRDS neonates who received PS replacement therapy at the 24th hour, the 48th hour, the 72nd hour, and the 7th day are presented in Table 6. After PS treatment, nLUS gradually decreased. The progression of lung lesion improvement after PS replacement therapy followed a front-to-back and top-to-bottom pattern. The patients first experiences a gradual reduction or disappearance of subpleural consolidation, followed by a decrease in B-lines and a gradual appearance of A-lines (see Figures 3, 4). Clinical symptoms improve, including hypoxia and cyanosis. The results of the analysis of variance showed that there was no significant difference (*P* = 0.78) between the 24th hours after receiving PS treatment and the 48th hours after receiving PS treatment using the lung twelve zone method, while there were significant differences (*P* < 0.05) in other nLUS. nLUS can better measure the efficacy of PS treatment, among which the pulmonary fourteen zone method can better reflect the early effects of PS replacement therapy.

**Table 6:** Dynamic monitoring of nLUS after PS replacement therapy.

	Time-histories	Score	*F*	*P* value
nLUS_12_	Before PS	21.36 (20.27, 22.45)	62.28	<0.01
	24 h	17.97 (16.87, 19.06)		
	48 h	16.36 (15.35, 17.37)		
	72 h	14.47 (13.45, 15.48)		
	7 days	10.42 (9.49, 11.36)		
nLUS_14_	Before PS	41.44 (39.92, 42.95)	121.25	<0.01
	24 h	38.48 (36.90, 40.07)		
	48 h	33.45 (31.85, 35.05)		
	72 h	27.91 (26.15, 29.66)		
	7 days	18.77 (17.05, 20.48)		

Values were median (interquartile range). nLUS_12_, 12-zone lung ultrasound method; nLUS_14_, 14-zone lung ultrasound method; PS, pulmonary surfactant.

## Discussion

This study evaluated the diagnostic accuracy of nLUS and CXR score in neonates with RDS and related trends of dynamic efficacy evaluation after PS replacement therapy and introduced the nLUS_14_ for comparative analysis. The results indicated that nLUS exhibits high accuracy in diagnosing NRDS, thereby enhancing the diagnosis value of LUS as a new non-invasive tool in NRDS (28–31). This study was strictly conducted by two sonographers with over 10 years of experience to ensure the accuracy and analyzability of the research results. The study found that nLUS and CXR scoring results demonstrate good consistency in the diagnosis of NRDS. Ultrasound can significantly reduce the possibility of malignant consequences caused by potential factors (e.g., long-term multiple movements, stress, etc.) during the examination process in neonates. Its technical requirements and experience reserves for operating physicians are not strict, which is conducive to the implementation of LUS in routine clinical practice at different levels of hospitals and improves the rapid diagnosis and treatment efficiency of NRDS.

Zhu et al. (32) found that nLUS positively correlates with the volume of pulmonary edema and the pathological severity of lung tissue. Blank et al. (33) demonstrated that complete airway fluid clearance cannot be accomplished in the first 4 h after delivery, healthy neonates being able to accomplish lung ventilation and alveolar fluid clearance in the first minute after birth. When using nLUS_12_ with an optimal diagnostic threshold of 13.5, the SEN, SPE, and ACC values are 0.94, 0.91, and 0.94, respectively. Pang et al. (34) used 21.5 as the diagnostic threshold, with a SEN of 0.80, SPE of 1.00, and ACC of 0.90. The optimal cut-off value of nLUS_14_ was 34.0 in this study, whereas the diagnostic threshold of nLUS_14_ in the experiment of Jiang et al. (33) was 41.0 points. This study differs slightly from other researchers' research, possibly because it delay LUS examination for some patients whose clinical manifestations are not critical. The alveoli are fully dilated and ventilated, and physiological alveolar and interstitial fluids are maximally cleared, reducing the interference of physiological tiny B-lines extending from the pleura to the deep lungs during ultrasound characterization.

Pang et al. (35) in their study of NRDS neonates with transient neonatal shortness of breath found that the LUS score had the best sensitivity and specificity for severe vs. mild/moderate NRDS using a cut-off value of 25.5, and Hua et al. (1) in a related study defined the optimal cut-off value for non-severe NRDS vs. severe NRDS to be 26.5. In the present study, we found that the optimal cut-off value for CXR grade III and CXR grade IV was 27.5, and when the cut-off value was 27.5, the LUS score SEN was 0.82, SPE was 0.45, ACC was 0.70, and AUC was 0.69 (0.50, 0.88). The relatively consistent cut-off values may indicate that CXR III and IV classification can be used as one of the criteria to judge severe vs. mild/moderate NRDS, and the fluctuation in the range of cut-off values may demonstrate the intersectionality of LUS in severe vs. mild/moderate imaging manifestations of NRDS. Among the misdiagnosed cases in this study, patients with pleural effusion accounted for a large proportion. In neonates lying supine in the incubator for a long period, a small amount of pleural fluid accumulates in the alveoli and interstitium in the posterior thorax and lung base due to gravity, and it is difficult for CXR to differentiate the small amount of fluid in the lung tissue in the surrounding area. The misdiagnosis of NRDS can be significantly reduced by using LUS, which has a very high sensitivity to aqueous density, for zonal scanning of the lungs, and in particular the nLUS_14_ for scanning the lung base alone.

LUS can assist in determining the nature of the pleural effusion which can be used to provide adjunctive support to the clinical trial data. The site of improvement of lung lesions after receiving PS replacement therapy shows an anterior-to-posterior, top-to-bottom order also correlates with gravitational deposition of effusion in the lungs (33). LUS can determine whether a child needs treatment in the neonatal intensive care unit and predict whether the child requires ventilator assistance and support (19). Exogenous PS replacement therapy via nasal continuous positive airway pressure conditions is an important treatment for NRDS. Surfactant entry into the alveoli results in a rapid reduction in alveolar surface tension, reconstruction of collapsed and atrophied alveoli, and gradual recovery of lung ventilation. In this study, we found that nLUS relative to the CXR score more accurately predicted the need for PS replacement therapy in neonates. Previous studies have suggested that the sensitivity and specificity of nLUS_12_ for pulmonary solid lesions are 0.90 and 0.98, respectively (3, 36, 37). In the early stages of PS treatment, the primary indication that patients with severe NRDS are gradually beginning to improve is the gradual reduction but not disappearance of the extent of pulmonary solid lesions. nLUS_14_ is more helpful for the control of severe NRDS patients' conditions, as it is refined into subpleural lung solid lesions and extensive lung solid lesions at a depth of one centimeter based on nLUS_12_ for the legal determination of the presence or absence of lung solid lesions (see Figures 3, 4). Although the dynamic observation of CXR was not performed in this study, it was confirmed that nLUS_14_ could better reflect the early effect after drug administration than nLUS_12_, and this result was consistent with the findings of Jiang et al. (33). Previous studies have confirmed the use of nLUS in identifying the cause of respiratory distress, the administration of surfactant or not, the time of drug withdrawal, the administration of mechanical ventilation, and the timing of withdrawal in neonates with NRDS (18, 19, 26, 38, 39). The present study refined some of the previous ideas and confirmed the value of LUS, especially nLUS_14_, in the diagnosis and treatment of patients with NRDS. However, there are still obvious limitations: firstly, the number of study cases included in this study was relatively small, and the reliability of the data needs to be verified by large-scale multicentre trials. This limits the validity and generalisability of the findings. Second, in view of subject health and ethical safety considerations, this study did not perform CXR examination during surfactant replacement sessions and did not compare the evaluation of the dynamic efficacy of nLUS and CXR on PS treatment. Given the above, our team will continue to carry out relevant studies to supplement the advancement of the study results at a later stage.

## Conclusion

LUS accurately detects NRDS in newborns presenting with rapid breathing. nLUS, particularly nLUS_14_, is reliable for determining whether NRDS patients need PS replacement therapy. Moreover, nLUS_14_ effectively assesses the early efficacy of PS replacement therapy, facilitating timely evaluation of treatment outcomes in infants with NRDS.

## Data Availability

The original contributions presented in the study are included in the article/Supplementary Material, further inquiries can be directed to the corresponding author.
